# Targeting the SphK1/S1P/PFKFB3 axis suppresses hepatocellular carcinoma progression by disrupting glycolytic energy supply that drives tumor angiogenesis

**DOI:** 10.1186/s12967-023-04830-z

**Published:** 2024-01-10

**Authors:** Xin Tracy Liu, Yu Huang, Da Liu, Yingxin Celia Jiang, Min Zhao, Long Hoa Chung, Xingxing Daisy Han, Yinan Zhao, Jinbiao Chen, Paul Coleman, Ka Ka Ting, Collin Tran, Yingying Su, Claude Vincent Dennis, Atul Bhatnagar, Ken Liu, Anthony Simon Don, Mathew Alexander Vadas, Mark Douglas Gorrell, Shubiao Zhang, Michael Murray, Mary Meltem Kavurma, Geoffrey William McCaughan, Jennifer Ruth Gamble, Yanfei Qi

**Affiliations:** 1 Centenary Institute of Cancer Medicine and Cell Biology, The University of Sydney, Sydney, NSW 2050 Australia; 2 School of Science, Technology and Engineering, University of the Sunshine Coast, Maroochydore DC, QLD 4558 Australia; 3 Key Laboratory of Biotechnology and Bioresources Utilization of Ministry of Education, Dalian Minzu University, Dalian, 116600 Liaoning China; 4 School of Medical Sciences, Faculty of Medicine and Health, The University of Sydney, Sydney, NSW 2006 Australia; 5 Sydney Microscopy and Microanalysis, The University of Sydney, Sydney, NSW 2006 Australia; 6 AW Morrow Gastroenterology and Liver Centre, Royal Prince Alfred Hospital, Sydney Local Health District, Sydney, NSW 2050 Australia; 7 Sydney Mass Spectrometry, The University of Sydney, Sydney, NSW 2006 Australia; 8 Sydney Pharmacy School, Faculty of Medicine and Health, The University of Sydney, Sydney, NSW 2006 Australia; 9 Heart Research Institute, Sydney, NSW 2042 Australia

**Keywords:** Sphingosine kinase, PF-543, PFKFB3, Angiogenesis, Glycolysis, Hepatocellular carcinoma

## Abstract

**Background:**

Hepatocellular carcinoma (HCC) remains a leading life-threatening health challenge worldwide, with pressing needs for novel therapeutic strategies. Sphingosine kinase 1 (SphK1), a well-established pro-cancer enzyme, is aberrantly overexpressed in a multitude of malignancies, including HCC. Our previous research has shown that genetic ablation of *Sphk1* mitigates HCC progression in mice. Therefore, the development of PF-543, a highly selective SphK1 inhibitor, opens a new avenue for HCC treatment. However, the anti-cancer efficacy of PF-543 has not yet been investigated in primary cancer models in vivo, thereby limiting its further translation.

**Methods:**

Building upon the identification of the active form of SphK1 as a viable therapeutic target in human HCC specimens, we assessed the capacity of PF-543 in suppressing tumor progression using a diethylnitrosamine-induced mouse model of primary HCC. We further delineated its underlying mechanisms in both HCC and endothelial cells. Key findings were validated in *Sphk1* knockout mice and lentiviral-mediated SphK1 knockdown cells.

**Results:**

SphK1 activity was found to be elevated in human HCC tissues. Administration of PF-543 effectively abrogated hepatic SphK1 activity and significantly suppressed HCC progression in diethylnitrosamine-treated mice. The primary mechanism of action was through the inhibition of tumor neovascularization, as PF-543 disrupted endothelial cell angiogenesis even in a pro-angiogenic milieu. Mechanistically, PF-543 induced proteasomal degradation of the critical glycolytic enzyme 6-phosphofructo-2-kinase/fructose-2,6-biphosphatase 3, thus restricting the energy supply essential for tumor angiogenesis. These effects of PF-543 could be reversed upon S1P supplementation in an S1P receptor-dependent manner.

**Conclusions:**

This study provides the first in vivo evidence supporting the potential of PF-543 as an effective anti-HCC agent. It also uncovers previously undescribed links between the pro-cancer, pro-angiogenic and pro-glycolytic roles of the SphK1/S1P/S1P receptor axis. Importantly, unlike conventional anti-HCC drugs that target individual pro-angiogenic drivers, PF-543 impairs the PFKFB3-dictated glycolytic energy engine that fuels tumor angiogenesis, representing a novel and potentially safer therapeutic strategy for HCC.

**Supplementary Information:**

The online version contains supplementary material available at 10.1186/s12967-023-04830-z.

## Introduction

Hepatocellular carcinoma (HCC) accounts for more than 90% of primary liver cancers, which is the third leading cause of cancer-related deaths worldwide [1]. HCC is one of the most vascularized solid tumors, in which aberrant neovascularization increases the access of tumors to blood and nutrient supplies, promoting cancer cell proliferation, immune cell infiltration, tumor growth and metastasis [2, 3]. Clinically, increased tumor angiogenesis is positively correlated with HCC tumor volume and aggressiveness [4]. Vascular endothelial growth factor A (VEGF-A) and its receptor VEGFR2 represent major tumor angiogenic mechanisms. Over 90% of advanced HCC cases exhibit significantly elevated VEGF-A levels [5]. Currently, therapeutic agents, such as sorafenib and bevacizumab, primarily target VEGF-A/VEGFR2 to impede HCC progression [6, 7]. However, blocking VEGF-A/VEGFR-2 does not adequately abrogate tumor angiogenesis due to the redundancy of other pro-angiogenic growth factors [5, 8]. As such, these agents show limited therapeutic effectiveness [9, 10]. Therefore, new anti-angiogenic strategies should be considered in HCC treatment.

Endothelial cells predominantly utilize glycolysis for energy production [11]. Notably, glycolysis is further increased by ~ 30% in tumor endothelial cells, which supports their sprouting, migration and tube formation during tumor neovascularization [12, 13]. These processes are dictated by the glycolytic activator 6-phosphofructo-2-kinase/fructose-2,6-biphosphatase 3 (PFKFB3) [11, 12]. Knockout (KO) or inhibition of PFKFB3 inhibits tumor angiogenesis, growth and metastasis, promotes tumor vessel normalization and improves chemotherapy, irrespective of VEGF-A presence [12, 14]. Targeting PFKFB3-mediated glycolytic energy supply for tumor angiogenesis represents a distinct, potentially superior anti-angiogenic strategy, compared to molecularly targeting individual pro-angiogenic drivers [8, 13]. This approach warrants further exploration in HCC therapeutics.

Sphingolipids are a class of essential lipids with extensive roles in cellular structure and signaling. As a rate-limiting enzyme in the catabolism of sphingolipids, sphingosine kinase 1 (SphK1) catalyzes the conversion of ceramide and sphingosine into sphingosine 1-phosphate (S1P) [15, 16]. SphK1 is aberrantly overexpressed in various cancers, including HCC, and correlates with poor prognoses [17–19]. SphK1 levels are elevated in a mouse model of HCC induced by diethylnitrosamine (DEN) [20], while germline *Sphk1*-KO suppresses HCC development in these mice [21]. It is noteworthy that SphK1 and S1P are critical regulators of angiogenesis. SphK1/S1P promotes angiogenesis in endothelial cells and matrigel plugs [22, 23]. In contrast, depletion of S1P impairs vascular development [24] and suppresses tumor growth in xenograft and allograft models by inhibiting VEGF-A-induced neovascularization [23]. Therefore, it is now important to investigate how SphK1/S1P promotes angiogenesis in endothelial cells and whether this regulation can be exploited for HCC treatment.

Given the well-documented pro-cancer role of SphK1, its highly selective inhibitor, PF-543, emerges as a promising candidate for cancer therapy [25]. PF-543 exhibits a notably low inhibition constant Ki of 3.6 nM against SphK1 in vitro [25], and it decreases S1P production in a range of cell types [25–28]. Intriguingly, PF-543 inhibits hepatocyte growth factor-induced sprouting angiogenesis in human lung microvascular endothelial cells [29]. To date, PF-543 has never been tested in any primary cancer models in vivo. In this study, we assessed the anti-angiogenic and anti-cancer effects of PF-543 in DEN-induced mouse HCC and elucidated its underlying anti-HCC mechanisms. Our study uncovered a previously unidentified pathway that links the SphK1/S1P/S1P receptor axis and PFKFB3-mediated glycolysis in the regulation of angiogenesis. It also demonstrated that pharmacological targeting of SphK1 offers a novel strategy for inhibiting tumor angiogenesis by disrupting glycolytic energy production, with potential significance for the development of effective HCC treatments.

## Materials and methods

### Human HCC specimens

Human HCC and adjacent para-tumorous tissue specimens were obtained from the Royal Prince Alfred Hospital (RPAH), Sydney, Australia. The sample collection was authorized by the Sydney Local Health District Human Research Ethics Committee (SLHD-HREC, #2019/ETH13790). All subjects provided informed consent prior to specimen acquisition.

### Animal work

Wild-type (WT) and *Sphk1*-KO mice on a C57BL/6J background were used in compliance with protocols (#2019-033 for PF-543 treatment and #2014-007 for *Sphk1*-KO) approved by the Research Ethics and Governance Office, RPAH. *Sphk1*-KO mice were obtained from Dr Richard Proia, National Institutes of Health, USA [30]. Mice were housed on a 12-hour light/dark cycle and were allowed food and water *ad libitum*. Male mice aged 12–14 days were intraperitoneally injected with DEN (Sigma-Aldrich) at 25 mg/kg body weight to induce liver tumors. PF-543 (Selleckchem) was initially dissolved in dimethyl sulfoxide and further diluted in a solvent consisting of 40% PEG-300, 5% Tween 80 and water. At 25 weeks of age, mice were randomly assigned to two groups and intraperitoneally injected with either PF-543 at 25 mg/kg or vehicle every other day for 12 weeks.

### Cell culture

Huh7 HCC cell line was obtained from and authenticated by CellBank Australia, human dermal microvascular endothelial cells (HMEC-1) were originally from the American Type Culture Collection, while primary human umbilical vein endothelial cells (HUVECs) were isolated from umbilical cords authorized by the SLHD-HREC (#2019/ETH06859). Cells were maintained in a 5% CO_2_ incubator using the previously detailed culture media [31–33]. Huh7 and HMEC-1 cells were tested mycoplasma-free. Short hairpin RNAs targeting SphK1 (#a or if not specified, 5ʹ-GCAGCTTCCTTGAACCATTAT-3ʹ; #b, 5ʹ-CGCTGTGCCTTAGTGTCTACT-3ʹ) were constructed in pLKO.1 lentiviral vector (Sigma-Aldrich). The lentivirus was generated in HEK293T cells using plasmids obtained from Dr Didier Trono through Addgene, including pMD2.G, pMDLg/pRRE and pRSV-Rev [34]. PF-543 and W146 used for cell culture were purchased from Cayman Chemical; MG-132 was from Calbiochem; VEGF-A was from R&D Systems; while D-erythro S1P was from Enzo Life Sciences.

### Lipidomics

Lipids were extracted using a 1:1 mixture of methanol and butanol containing a panel of internal lipid standards (Avanti^®^ Polar Lipids). Sphingolipids were quantified using targeted lipidomics on a TSQ Altis triple quadrupole mass spectrometer, following lipid separation using an Agilent Eclipse Plus C8 column on a Vanquish^™^ ultra high-performance liquid chromatography system [35]. Peaks were integrated using Xcalibur [35]. Absolute concentrations were determined by normalization against corresponding internal standards and tissue weight. Hierarchical clustering was employed to group similar patterns of lipid changes using Euclidean distance and Ward’s algorithm, where Z-scores were calculated to measure the relative deviation of individual lipid concentrations from the mean values (Metaboanalyst 5.0).

### Tissue staining

Liver histology was examined by hematoxylin and eosin (H&E) staining [33]. Immunohistochemistry (IHC) and immunofluorescent (IF) staining were conducted on paraffin-embedded sections and cryosections, respectively, using antibodies against SphK1 (Santa Cruz Biotechnology), phospho-SphK1 (Proteintech), Ki67 (Abcam), CD31 (CST for IHC and BD Pharmingen for IF), cleaved caspase-3 (Cell Signaling Technology, CST) and PFKFB3 (Proteintech). Brightfield Images were captured using Nikon Ni-E Widefield and Zeiss Axio Scan Z1 microscopes, while IF images were obtained on a Nikon C2 confocal microscope. Images were quantified using ImageJ.

### Cell viability and cell death

Cell viability was determined by MTS assay (Promega). Luminescence was measured at 490 nm using a TECAN Infinite M1000 Pro plate reader. Cell death was examined following propidium iodide staining on a BD LSRII (BD™ Biosciences) flow cytometer [36]. Data analysis was performed using FlowJo.

### Colony formation

Huh7 cells were treated with PF-543 for 10 days. The medium containing PF-543 was refreshed every 3 days. Colonies were fixed using 4% cold paraformaldehyde and stained with 0.5% w/v crystal violet [37]. Images were captured using a ChemiDoc^™^ Touch Imaging System (Bio-Rad Laboratories) and quantified using ImageJ.

### Endothelial cell migration

Cells were seeded in the upper chambers of transwell inserts with 8 μm-pore polycarbonate membrane filter (Sigma-Aldrich). After 12 h of culture, migrated cells in the lower chambers were fixed with 4% paraformaldehyde and stained with 0.5% w/v crystal violet for counting.

### Endothelial cell tube formation

Cells were seeded onto polymerized growth factor reduced matrigel (Sigma-Aldrich) in 96-well plates, containing 50 ng/ml VEGF-A or a conditioned medium collected from 72 h culture of Huh7 HCC cells. Images of vessel-like tubular network formation were captured at 6–8 h using a Nikon Ti-E Spinning Disk microscope and analyzed using the *Angiogenesis* plugin in ImageJ.

### Endothelial cell sprouting

HUVEC spheroids were allowed to form in a medium containing 2% methylcellulose (Sigma-Aldrich), before being transferred to plates coated with a mixture of collagen I (Sigma-Aldrich) and methylcellulose [38]. Following the treatment, spheroids were fixed in 4% paraformaldehyde, imaged on a Nikon Ti-E Spinning Disk microscope and quantified using ImageJ.

### Pathway commons analysis

To assess the associations between glycolytic and angiogenic (represented by VEGF signaling) pathways, we downloaded 128 relevant genes from the KEGG database, including *PFKFB3* and *SPHK1*. Utilizing the GenRev algorithm, we extracted a subnetwork from the human interactome, sourced from the Pathway Commons database (www.pathwaycommons.org*).* This database was selected for its comprehensive and reliable interaction data, minimizing noise and bias. The subnetwork reflected the shortest pathway linkages among the selected genes within the interactome.

### Western blotting

Proteins were extracted from mouse liver tissues and HUVECs [33]. Immunoblotting was conducted using antibodies against p-VEGFR2, t-VEGFR2, glucose transporter 1 (GLUT1), caspase-3, ubiquitin, GAPDH and β-actin from CST, SphK1 from Santa Cruz Biotechnology, CD31 from Abcam as well as phospho-SphK1, SphK2 and PFKFB3 from Proteintech. Chemiluminescence was detected using a Bio-Rad ChemiDoc^™^ Touch Imaging System.

### Quantitative RT-PCR analysis

RNA was extracted using TRIZol (Thermo Fisher), and reverse transcription was performed using a High-Capacity cDNA Reverse Transcription Kit (Thermo Fisher), as previously described [39]. PCR was conducted on a Roche Lightcycler 480 machine using SensiFAST^™^ SYBR Lo-ROX Kit (Bioline). Primer sets used for PCR were human *PFKFB3* F-*CAGTTGTGGCCTCCAATATC*, R-*GGCTTCATAGCAACTGATCC* [40]; human *β-ACTIN* F-*CATGTACGTTGCTATCCAGGC*, R- *CTCCTTAATGTCACGCACGAT* [41].

### Seahorse glycolysis assay

Glycolysis was determined using the Seahorse XF Glycolysis Stress Test Kit (Agilent) on an XFe96 analyzer. After the treatment, cells were seeded in collagen-precoated 96-well microplates. Extracellular acidification rates were measured by serial injections of glucose, oligomycin and 2-deoxy-D-glucose.

### Statistics

Comparisons between two groups were analyzed by unpaired two-tailed *t*-tests, and multiple comparisons were analyzed by one-way ANOVA with Tukey tests, using GraphPad Prism (version 9.5). Differences at values of *p* < 0.05 were considered significant.

## Results

### PF-543 inhibits SphK1 activity in DEN-treated mice

**Fig. 1:**
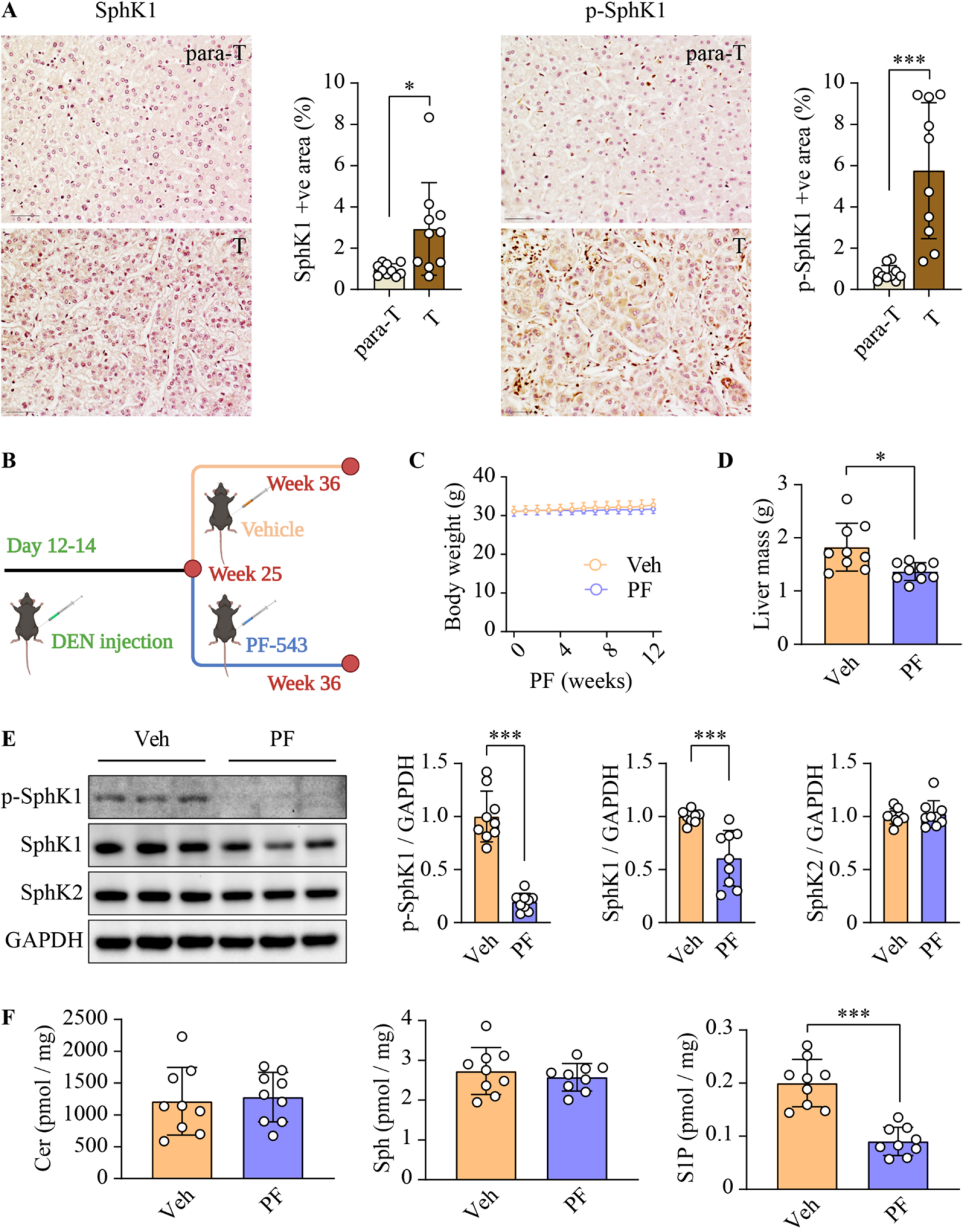
PF-543 inhibits SphK1 activity in DEN-treated mice.** A** Total and phospho-SphK1 levels were examined using immunohistochemical staining in tumorous (T) and para-tumorous (para-T) tissues of human HCC specimens; scale bar = 50 μm; n = 10. **B** Schematic illustration of treatment schedules for in vivo studies, created with BioRender.com. **C** Body weight over 12 weeks of vehicle (veh) or PF-543 (PF) treatment. **D** Liver mass was weighed. **E** phospho(p)-SphK1 SphK1 and SphK2 protein levels in liver tissues were determined using Western blotting. **F** Hepatic levels of ceramide (Cer), sphingosine (Sph), and S1P were analyzed using lipidomics. **C-F** n = 9. Data are expressed as mean ± SD. *p < 0.05; ***p < 0.001

Consistent with previous findings [21], SphK1 expression was increased in human HCC relative to adjacent non-tumorous tissues (Fig. 1A). To further elucidate the functional implications of the increased SphK1 activity in human HCC, we quantified the expression of its phosphorylated, active form - phospho-SphK1-S225 [42]. We observed a 7-fold upregulation of phospho-SphK1-S225 in human HCC tissues (Fig. 1A). To assess the therapeutic potential of SphK1 inhibition, we induced HCC-like liver cancer in mice with a single injection of DEN. Mice were then treated with PF-543 or vehicle on alternate days for 12 weeks, commencing at 25 weeks of age when initial neoplastic lesions were established (Fig. 1B). Body weights remained comparable between the two groups throughout the study (Fig. 1C). Notably, PF-543 significantly reduced liver mass by 25.2% (Fig. 1D). To ascertain the in vivo inhibitory effects of PF-543 on SphK1 activity, we examined the levels of phospho-SphK1 and its enzymatic product S1P, 24 h post-final PF-543 injection. PF-543 decreased SphK1 phosphorylation and total protein expression by 80% and 40%, respectively, in the liver, without affecting SphK2 (Fig. 1E). In addition, we assessed sphingolipidomic alterations following PF-543 treatment in the liver. Among 69 identified sphingolipid species, PF-543 specifically reduced hepatic S1P levels by 54.9% (*p* < 0.001) (Additional file 1: Fig. S1 and Fig. 1F). In line with these observations, PF-543 also markedly decreased plasma S1P levels by 50% (Additional file 1: Fig. S2). These findings indicate that effective SphK1 inhibition was sustained across the dosing intervals. In contrast, PF-543 had minimal impact on ceramide and sphingosine levels (Fig. 1F and Additional file 1:  Figs. S1, S2).

### Inhibition of SphK1 suppresses HCC progression in DEN-treated mice

**Fig. 2:**
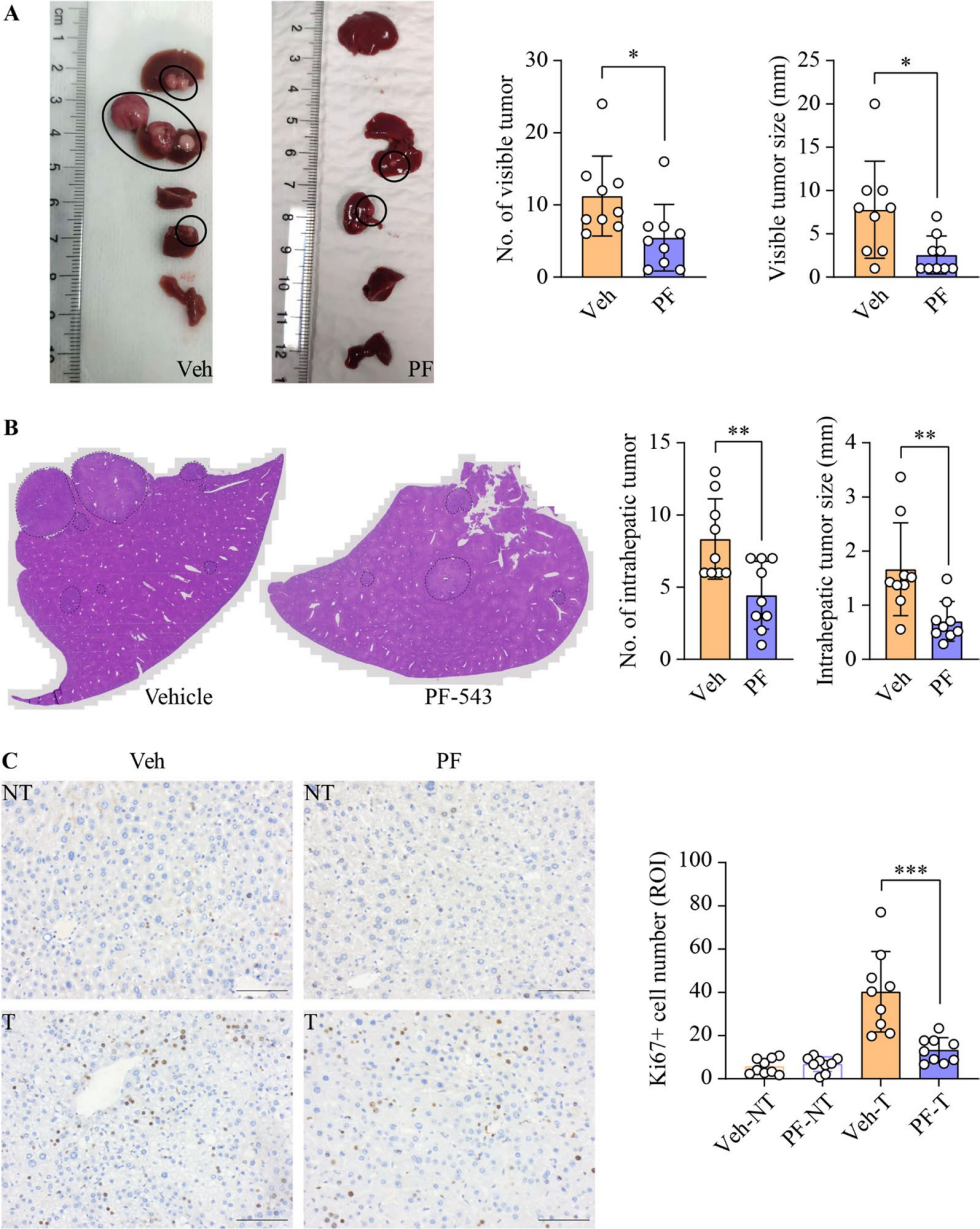
Inhibition of SphK1 suppresses HCC progression in DEN-treated mice. DEN-injected mice were treated with vehicle (veh) or PF-543 (PF) for 12 weeks. **A** The number and maximal diameter of visible liver tumors were quantified from macroscopic images. **B** The number and maximal diameter of intrahepatic liver tumors were quantified from the scanning of the entire H&E-stained liver tissue sections. **C** Cell proliferation in non-tumorous (NT) and tumorous (T) liver tissues was stained and quantified using Ki67 immunohistochemistry; scale bar = 50 μm. Data are expressed as mean ± SD. n = 9. *p < 0.05; **p < 0.01; ***p < 0.001

Subsequently, we assessed the anti-HCC efficacy of PF-543 in DEN-treated mice. Remarkably, PF-543 treatment led to a 51% reduction in the number of macroscopically visible tumors and a 67% decrease in maximal tumor size (Fig. 2A). PF-543 also significantly decreased intrahepatic tumor number and size by 47% and 58%, respectively (Fig. 2B). In line with these findings, immunohistochemical analysis revealed a 3-fold reduction in the proportion of cells positive for the proliferation marker Ki67 in liver tumors from PF-543-treated mice, as compared to vehicle controls (Fig. 2C). To further understand the anti-HCC mechanisms of PF-543, we examined its anti-cancer properties in Huh7 HCC cells. Consistent with in vivo data, PF-543 markedly reduced S1P levels by 68% in Huh7 cells (Additional file 1: Fig. S3A). Intriguingly, despite its evident anti-proliferative effects in vivo, PF-543 did not affect HCC cell viability in vitro (Additional file 1:  Fig. S3B). In addition, PF-543 neither induced cell death nor suppressed clonogenicity (Additional file 1:  Fig. S3C and S3D). Furthermore, PF-543 did not induce caspase-3 cleavage in liver tissues, indicating that apoptosis was not activated (Additional file 1: Fig. S4). Collectively, these data suggest that the anti-HCC actions of PF-543 observed in vivo were not mediated through direct cytotoxicity on HCC cells.

### SphK1 inhibition or ablation reduces vessel density in HCC

**Fig. 3:**
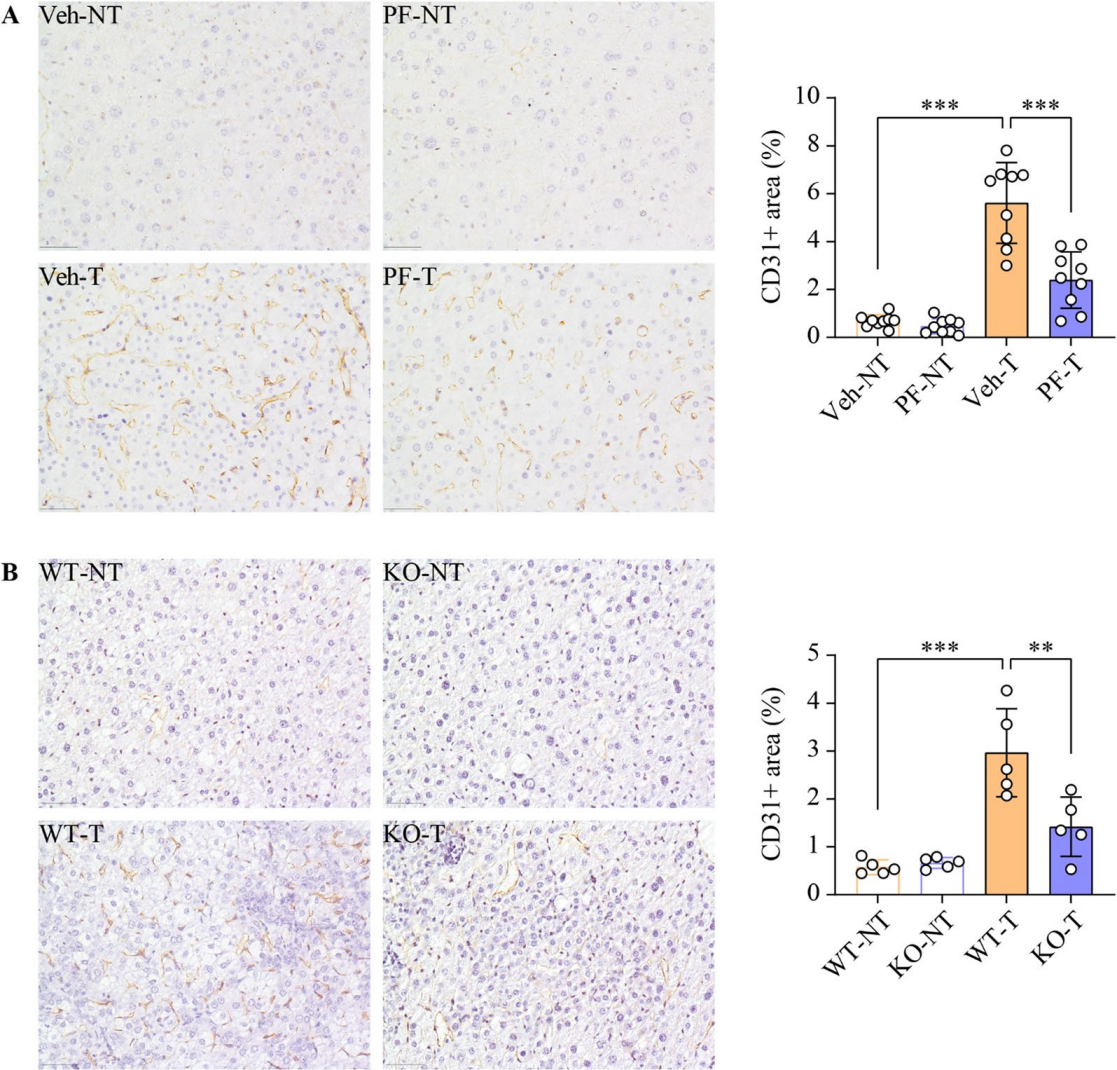
SphK1 inhibition or ablation reduces vessel density in HCC tumors. **A** DEN-injected mice were treated with vehicle (veh) or PF-543 (PF) for 12 weeks. Blood vessels in non-tumorous (NT) and tumorous (T) liver tissues were stained and quantified by CD31 immunohistochemistry; scale bar = 50 μm; n = 9. **B** Blood vessels in DEN-injected wild-type (WT) and *Sphk1* knockout (KO) mice were examined by CD31 immunohistochemistry; scale bar = 50 μm; n = 5. Data are expressed as mean ± SD. **p < 0.01; ***p < 0.001

Given the critical role of tumor neovascularization in HCC progression and the pro-angiogenic effects of SphK1, we examined the impacts of PF-543 on intratumoral vessel density in DEN-induced HCC. The vessel density in liver tumors, as indicated by CD31 positive area, was 8.2 times greater than that in the adjacent non-tumorous tissues in the vehicle-treated group, demonstrating marked tumor neovascularization (Fig. 3A). Meanwhile, PF-543 treatment resulted in a 57% reduction in this elevated vessel density (Fig. 3A). To determine whether this anti-angiogenic effects of PF-543 could be attributed specifically to SphK1 inhibition, we conducted a parallel study using *Sphk1*-KO mice. Genetic ablation of *Sphk1* led to a 52% reduction in intratumoral vessel density (Fig. 3B), an effect comparable to that observed with PF-543 treatment. Importantly, PF-543 did not directly alter CD31 expression in endothelial cells (Additional file 1:  Fig. S5). These findings indicate a pivotal role of SphK1 in promoting HCC tumor neovascularization and suggest that targeting SphK1 represents a viable strategy for anti-HCC therapy through the modulation of angiogenesis.

### Inhibition of SphK1 impairs angiogenesis in endothelial cells

**Fig. 4:**
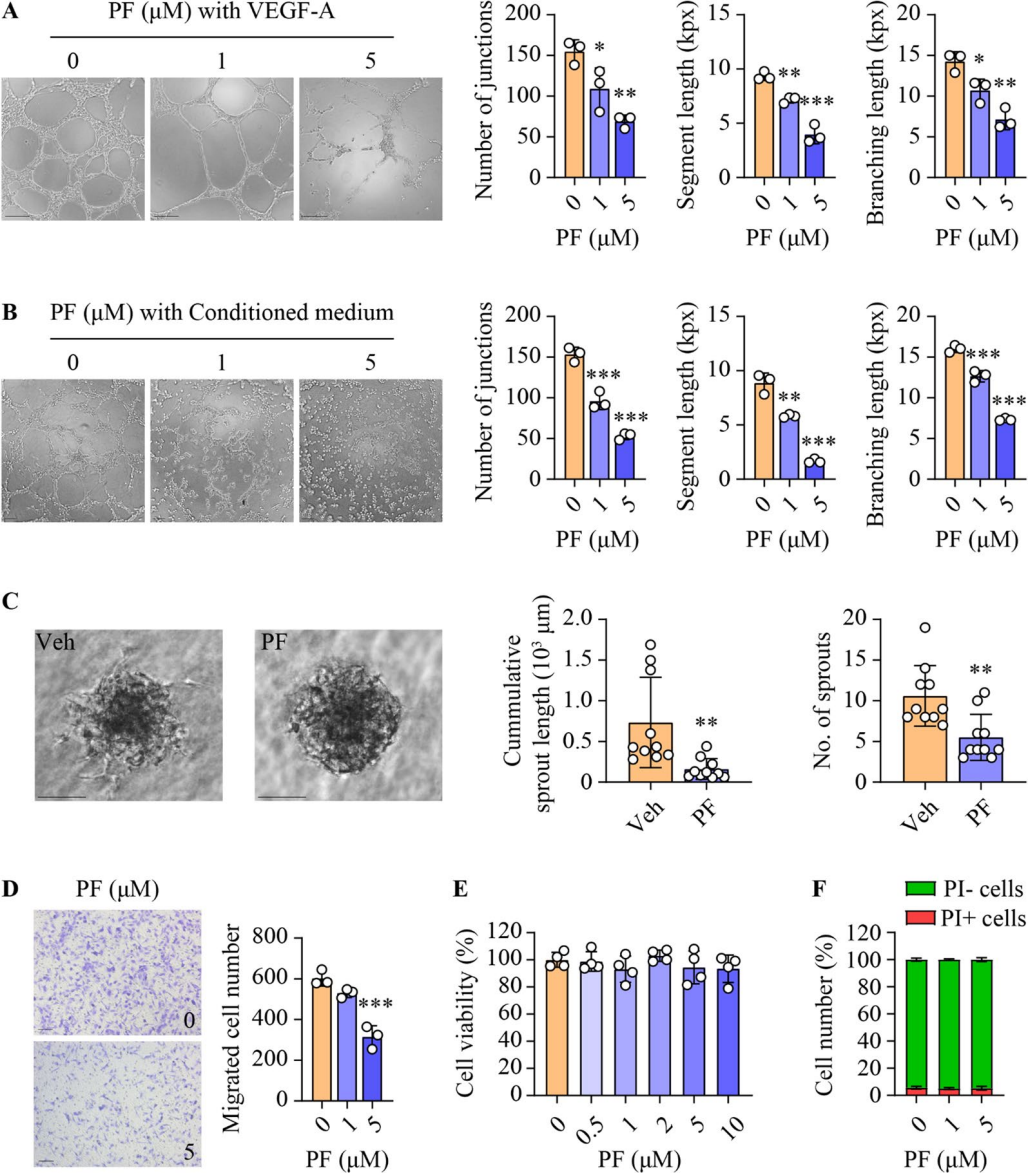
Inhibition of SphK1 impairs angiogenesis in HUVECs. Primary HUVECs were treated with PF-543 at the indicated concentrations for 16 h. **A** and **B** Tube formation was induced by 50 ng/ml VEGF-A (**A**) or conditioned medium collected from Huh7 HCC cell culture (**B**). Quantification of tube formation is presented as the number of junctions, segment length, and total branching length. kpx, 1000 pixels; scale bar = 200 μm (**A**) or 100 μm (**B**); n = 3. **C** Sprouting assays were performed in three-dimensional spheroids. Quantitation of the sprouting is presented as cumulative sprout length and number of sprouts per spheroid; scale bar = 100 μm; n = 10. **D** Cell migration was determined by transwell assay, and migrated cells were stained with crystal violet; scale bar = 100 μm; n = 3. **E** Cell viability was determined using MTS assay; n = 4. **F** Cell death was assessed using flow cytometry with propidium iodide (PI) staining; PI-, living cells (green); PI+, dead cells (red); n = 3. Data are expressed as mean ± SD. *p < 0.05; **p < 0.01; ***p < 0.001

To further delineate the anti-angiogenic actions of PF-543, we examined its impacts on various angiogenic parameters in both HUVEC and HMEC-1 endothelial cells. Upon stimulation with VEGF-A, PF-543 significantly decreased key metrics of endothelial tube formation, including the number of junctions, segment lengths and branching lengths, in a concentration-related fashion in both endothelial cell types (Fig. 4A and Additional file 1:  Fig. S6A). In addition, PF-543 also mitigated tube formation in HUVECs exposed to conditioned medium derived from Huh7 HCC cells (Fig. 4B), thereby suggesting a link between its anti-angiogenic and anti-HCC actions observed in vivo. In support, PF-543 markedly inhibited sprouting in HUVEC spheroids, leading to a 78% and 48% reduction in cumulative sprout length and the number of sprouts, respectively (Fig. 4C). Furthermore, the compound significantly attenuated the migratory capabilities of both HUVEC and HMEC-1 cells (Fig. 4D and Additional file 1:  Fig. S6B). Notably, PF-543 did not affect cell proliferation or induce cell death (Fig. 4E, F and Additional file 1:  Fig. S6C, D). Together, these findings implicate SphK1 as a key regulator of angiogenic processes in endothelial cells.

#### SphK1 promotes angiogenesis by regulating PFKFB3

**Fig. 5:**
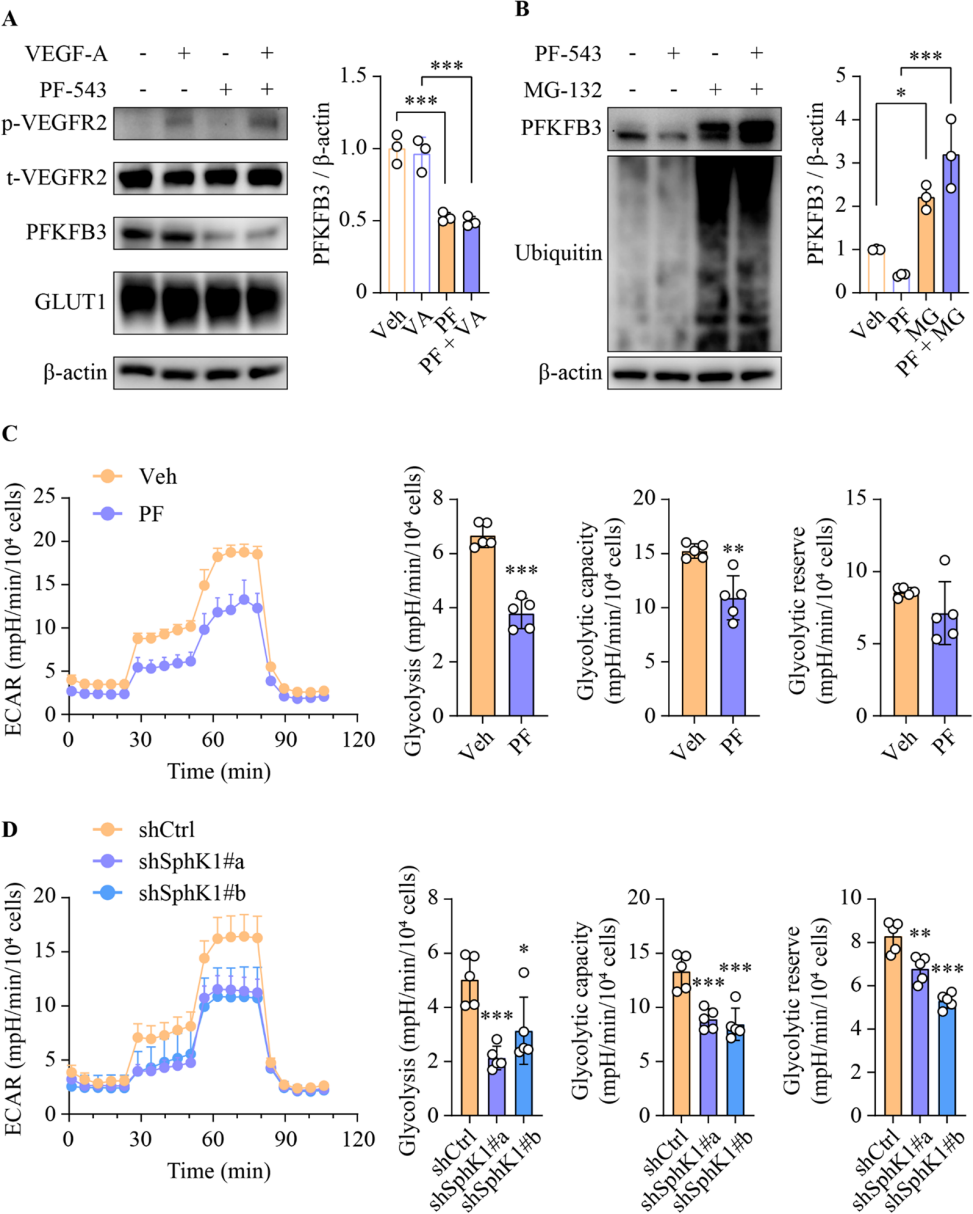
SphK1 promotes angiogenesis by regulating the glycolytic modulator PFKFB3. **A** Primary HUVECs were treated with 5 µM PF-543 for 16 h, prior to stimulation with 50 ng/ml VEGF-A for 15 min. **B** Following 1 h pre-treatment with 10 µM MG-132, primary HUVECs were treated with PF-543 at 5 µM for 16 h. **A** and **B** Western blotting analyses of the indicated proteins; n = 3. **C** and **D** Glycolytic rate, capacity and reserve were determined using Seahorse real-time glycolytic stress assay in PF-543-treated (**C**) or shRNA-mediated SphK1 knockdown (**D**) primary HUVECs. ECAR, extracellular acidification rate; n = 5. Data are expressed as mean ± SD. *p < 0.05; **p < 0.01; ***p < 0.001

Pathway commons analysis revealed a complex subnetwork comprising 1347 interactions among 128 genes relating to VEGF signaling and glycolysis pathways, including *SPHK1* (Additional file 1:  Fig. S7). This intricate web of connections suggested a potential role for SphK1 in the regulation of angiogenesis via glycolysis. To address this, we evaluated the impacts of PF-543 on key angiogenic and glycolytic regulators in HUVECs. Notably, PF-543 selectively downregulated the expression of PFKFB3, without affecting the levels of phospho-VEGFR2, total VEGFR2 or GLUT1, (Fig. 5A). This was also observed in DEN-treated mice, where elevated PFKFB3 levels in tumor endothelial cells and adjacent tumorous tissues were dramatically attenuated by PF-543 administration (Additional file 1:  Fig. S8). Interestingly, PFKFB3 mRNA levels were paradoxically increased in HUVECs following PF-543 treatment (Additional file 1:  Fig. S9), thereby ruling out transcriptional regulation. Instead, the PF-543-mediated reduction in PFKFB3 protein expression was abrogated by pre-treatment of cells with the proteasomal inhibitor MG-132, suggesting that PF-543 promoted the proteasomal degradation of PFKFB3 (Fig. 5B). In line with the pivotal role of PFKFB3 in glycolytic regulation, PF-543 treatment significantly decreased both glycolytic rate and capacity in VEGF-A-stimulated HUVECs (Fig. 5C). A similar glycolytic inhibition was observed upon SphK1 knockdown (Fig. 5D). Taken together, these findings unveil a hitherto unidentified functional interplay between SphK1 and PFKFB3 in the regulation of endothelial cell glycolysis, which PF-543 can modulate.

### SphK1 promotes glycolysis and angiogenesis through S1P in a receptor-dependent manner

**Fig. 6:**
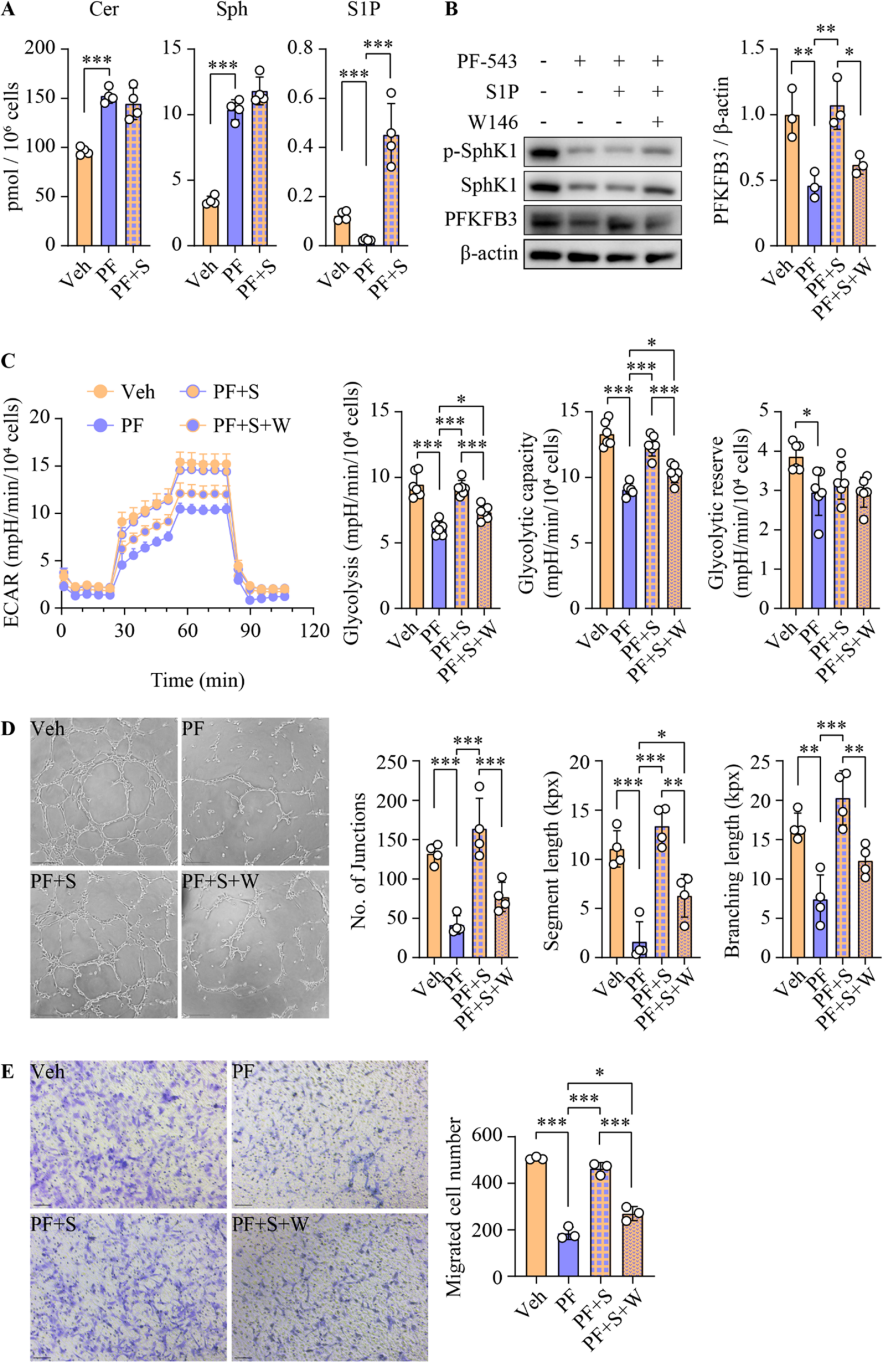
S1P is required for glycolysis and angiogenesis in an S1P_1_-dependent manner. Primary HUVECs were treated with 5 µM PF-543 (PF) and 0.5 µM S1P (S) for 16 h, with 2 µM S1P_1_ antagonist W146 added 1 h prior to these treatments. **A** Levels of ceramide (Cer), sphingosine (Sph) and S1P were analyzed using lipidomics; n = 4. **B** phospho(p)-SphK1, SphK1 and PFKFB3 protein levels were determined using Western blotting; n = 3. **C** Glycolytic rate, capacity and reserve were examined using Seahorse real-time glycolytic stress assay. ECAR, extracellular acidification rate; n = 6. **D** Tube formation was quantified as the number of junctions, segment length, and total branching length. kpx, 1000 pixels; scale bar = 200 μm; n = 4. **E** Cell migration was determined by transwell assay, and migrated cells were stained with crystal violet; scale bar = 100 μm; n = 3. Data are expressed as mean ± SD. *p < 0.05; **p < 0.01; ***p < 0.001

We also identified a 6.1-fold increase in PFKFB3 expression in human HCC relative to paratumorous tissues (Additional file 1:  Fig. S10A). Interestingly, PFKFB3 expression was significantly correlated with that of phospho-SphK1, but not total SphK1, among individuals (Additional file 1:  Fig. S10B). This suggests that S1P production by SphK1 is critical in the regulation of PFKFB3 expression and its associated functions. To elucidate this, we supplemented PF-543-treated HUVECs with exogenous S1P. PF-543 alone led to an 80.5% reduction in intracellular S1P levels, which was reversed upon S1P supplementation (Fig. 6A). In contrast, S1P treatment did not alter cellular levels of other sphingolipids, such as sphingosine and ceramide (Fig. 6A). Under these conditions, S1P supplementation effectively reversed PFKFB3 expression, without altering the levels of phosphorylated or total SphK1 (Fig. 6B). Importantly, the addition of S1P restored glycolytic rate and capacity and also normalized tube formation and cell migration, despite the ongoing SphK1 inhibition by PF-543 (Fig. 6C–E). Finally, we examined whether S1P-mediated functional restoration was receptor-dependent. S1P_1_ is the predominant S1P receptor isoform implicated in angiogenesis [43]. Blocking S1P_1_ with its specific antagonist W146 abrogated the S1P-mediated restoration of PFKFB3 levels, glycolysis, tube formation and cell migration in PF-543-treated HUVECs (Fig. 6B–E). Collectively, these findings strongly indicate that SphK1-derived S1P, rather than SphK1 itself, played a critical role in modulating PFKFB3-mediated glycolytic energy supply for angiogenesis, and this regulation was S1P_1_-dependent.

## Discussion

**Fig. 7:**
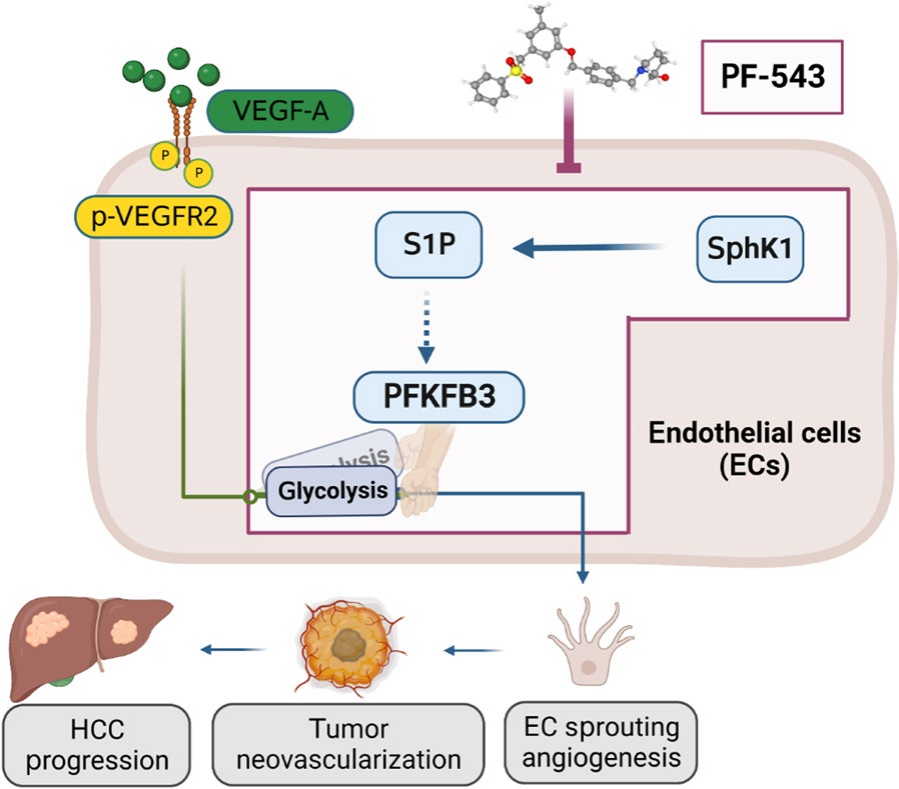
Model depicting the anti-HCC actions of PF-543. Elevated pro-angiogenic factors, exemplified by VEGF-A, act through their receptors to drive sprouting angiogenesis in tumor endothelial cells (EC), promoting tumor neovascularization and HCC progression. PFKFB3 serves as a molecular switch in this process, dictating the glycolytic energy supply that is essential for sprouting angiogenesis. The selective SphK1 inhibitor PF-543 abrogates S1P production, subsequently turning off the PFKFB3-mediated glycolytic switch, which leads to the inhibition of sprouting angiogenesis and eventually suppression of HCC progression. The graphical abstract was created with BioRender.com. . The 3D conformer of PF-543 was adapted from PubChem, *pubchem.ncbi.nlm.nih.gov*, CID 66,577,038

The therapeutic potential of PF-543 has previously been demonstrated in xenograft models of several cancer types, including colorectal cancer, triple-negative breast cancer and large-cell lung carcinoma [44–46]. However, its efficacy in primary cancer models remains unexplored. This study showed the anti-cancer effects of PF-543 against DEN-induced primary liver cancer in mice and also elucidated the underlying mechanisms (Fig. 7). We initiated PF-543 treatment during the liver tumor growth phase in mice, commencing at 23 weeks post-DEN injection, to specifically assess its effects on HCC progression rather than on lesion initiation. This approach is closely aligned with current clinical practice for HCC treatment. It is worth noting that a reduction in tumor growth by > 50% in animal models is generally considered to be an indicator of potential clinical eligibility for new therapeutics [47]. In our study, PF-543 treatment effectively reduced visible and intrahepatic tumor size by 67% and 58%, respectively (Fig. 2), meeting this criterion. In line with this, our previous research has shown that *Sphk1*-KO results in a comparable 60% reduction in tumor size during the tumor growth phase following DEN administration [21]. These findings, taken together, indicate a critical role of SphK1 in HCC progression and affirm the therapeutic promise of targeted SphK1 inhibition.

Increased secretion of VEGF-A by malignant hepatocytes drives tumor angiogenesis and thus contributes to HCC progression and poor prognosis [4, 5, 48]. Consequently, anti-VEGF-A agents, such as sorafenib and bevacizumab, are clinically used to treat HCC [6, 7]. However, both exhibit significant serious adverse events in approximately one-third of recipients [10]. Our study demonstrated PF-543 as a promising alternative. PF-543 impaired angiogenesis in endothelial cells, even in the presence of VEGF-A or conditioned medium from HCC cells (Fig. 4 and Additional file 1: Fig. S6). These in vitro findings were mirrored in vivo, where PF-543 profoundly reduced intratumoral vessel density in a pro-angiogenic environment (Fig. 3). Importantly, PF-543 achieved this without altering vessel density in non-tumorous liver tissues (Fig. 3) or compromising endothelial cell viability (Fig. 4 and Additional file 1: Fig. S6). Furthermore, PF-543 exhibited potent anti-HCC effects, but with minimal cytotoxicity in both normal and HCC liver cells (Fig. 2 and Additional file 1: Figs. S3, S4). These findings collectively suggest that PF-543 could offer a more targeted and potentially safer anti-angiogenic strategy for HCC treatment.

In healthy adults, endothelial cells remain quiescent. However, upon exposure to elevated levels of pro-angiogenic growth factors, as commonly seen in HCC tumors, endothelial cells are rapidly activated, leading to sprouting angiogenesis [49]. This process demands additional energy and relies on PFKFB3-mediated glycolysis [11, 12]. Previous studies have established that knockdown or inhibition of PFKFB3 suppresses both sprouting angiogenesis and HCC tumor growth, downstream of pro-angiogenic factors [11, 12, 14]. In the present study, PFKFB3 expression was markedly increased in endothelial cells of mouse HCC (Additional file 1: Fig. S8), which is consistent with recent findings in human HCC [14]. The PFKFB3 protein has a relatively short half-life of less than 4 h. Its stability is modulated by multiple post-translational mechanisms, including ubiquitination mediated by anaphase-promoting complex/cyclosome and SKP1/CUL-1/F-box [50, 51], deubiquitination by ubiquitin-specific protease 19, ubiquitin-specific protease 33 and OTU deubiquitinase 4 [52–54], methylation by protein arginine methyltransferases 1 and 4) [55], demethylation by lysine demethylase 2 A [56] and phosphorylation (e.g. murine lymphomas 2) [57]. These modifications collectively influence PFKFB3 protein’s stability and its degradation via the proteasomal pathway [50–57]. In the present study, PF-543 treatment significantly decreased PFKFB3 protein levels, which was reversed by the proteasomal inhibitor MG-132 (Fig. 5), indicating that PF-543 induced proteasomal degradation of PFKFB3 protein. Similarly, the PF-543-induced downregulation of PFKFB3 protein was restored by S1P supplementation (Fig. 6), suggesting a critical role for S1P in the regulation of PF-543-induced PFKFB3 protein degradation. Importantly, alterations in S1P levels, PFKFB3 protein expression and endothelial cell glycolysis were closely linked (Fig. 6), unveiling novel mechanistic and functional connections between S1P and PFKFB3. In contrast to its effects on PFKFB3 protein and functional glycolysis, PF-543 increased PFKFB3 mRNA levels (Additional file 1: Fig. S9). In addition, when proteasomal degradation was inhibited, PF-543 treatment strongly increased PFKFB3 protein expression (Fig. 5). This observation was in line with the noted increase in PFKFB3 mRNA levels, suggesting a transcriptional activation. It is well-documented that the regulation of PFKFB3 mRNA is even more complex. Transcriptional activation of the *PFKFB3* gene is dictated by a broad range of transcription factors, such as specificity protein 1, activating protein 2, progesterone receptor, nuclear factor 1, estrogen receptor, hypoxia-inducible factor 1α, nuclear factor-κB and yes-associated protein 1, that target an extensive 3500 bp region upstream from the transcription start site [58–60]. In view of this regulatory complexity, future studies could be undertaken to address the specific mechanisms by which S1P regulates PFKFB3 mRNA and protein in greater detail.

We previously proposed three modes of action for SphK: the S1P receptor-dependent mode, the intracellular partner mode and the substrate depletion mode [15]. In the present study, exogenous S1P supplementation in HUVECs reversed the PF-543-induced inhibition of glycolysis and angiogenesis, when ceramide and sphingosine levels remained unchanged (Fig. 6). This indicates that PF-543 primarily acted via S1P depletion rather than ceramide or sphingosine accumulation, thus ruling out substrate depletion as the mode of action. Interestingly, the addition of S1P failed to recover SphK1 phospho-activation or expression (Fig. 6), which negates the possibility that PF-543 exerted its effects through intracellular partners of SphK1. To further dissect whether PF-543 functioned through an S1P receptor-dependent mode, we co-treated cells with the selective S1P_1_ antagonist W146. Of the five S1P receptor isoforms, S1P_1_ is predominantly involved in vascular development and function [43]. Both global and endothelial cell-specific ablation of S1P_1_ leads to defective vascular development in mouse embryos [61, 62]. Here, we revealed that W146 significantly abrogated the S1P-mediated restoration of glycolysis and angiogenesis in PF-543-treated HUVECs (Fig. 6). This is the first evidence that implicates an S1P receptor, specifically S1P_1_, in the regulation of PFKFB3 expression and glycolysis in endothelial cells. In line, S1P increases glycolysis in osteosarcoma cells via S1P_3_, the predominant S1P receptor isoform in these cancer cells [63]. Our findings reinforce the notion that S1P modulates sprouting angiogenesis via an “inside-out” signaling mechanism [64]. However, the precise mechanism by which S1P/S1P_1_ modulates PFKFB3 in endothelial cells remains to be elucidated. A deeper understanding of this regulation could potentially enhance the management of various neovascular conditions, not limited to HCC [13].

In summary, the present study provides both experimental and mechanistic evidence demonstrating the therapeutic potential of the selective SphK1 inhibitor PF-543 in HCC. The anti-HCC effects of PF-543 were primarily attributed to the suppression of tumor angiogenesis rather than direct cytotoxicity on HCC cells. Mechanistically, PF-543 treatment reduced S1P production in endothelial cells, which impaired the PFKFB3-mediated glycolytic energy supply for tumor angiogenesis. This represents a significant paradigm that is distinct from the current growth factor-centric anti-angiogenic strategy in HCC treatment. These newly identified anti-HCC actions of PF-543, along with its apparent safety profile, support further preclinical and clinical evaluations in HCC.

### Supplementary Information

**Additional file 1.** **Supplemental Figure 1.** PF-543 specifically reduces hepatic S1P levels in DEN-treated mice. Hierarchical clustering heatmap contains all identified sphingolipid species. The clustering dendrogram was determined based on the Euclidean distances between lipid variables using the Ward clustering method. Color keys indicate standardized lipid concentrations in Z-scores, with red denoting higher and blue denoting lower concentrations. ***, *p* < 0.001; n = 6. **Supplemental Figure 2.** PF-543 decreases plasma S1P levels in DEN-treated mice. Plasma levels of ceramide (Cer), sphingosine (Sph), and S1P were analyzed using lipidomics. Data are expressed as mean ± SD; n = 9. ***p < 0.001. **Supplemental Figure 3.** PF-543 exhibits minimal cytotoxicity in Huh7 HCC cells. **A** Huh7 cells were treated with 5 μM PF-543 (PF) for 16 h, prior to the measurement of ceramide (Cer), sphingosine (Sph) and S1P levels by lipidomics; n = 4. **B** Cell viability was determined using MTS assay; n = 6. **C** Cell death was assessed using flow cytometry with propidium iodide (PI) staining; PI-, living cells (green); PI+, dead cells (red); n = 3. **D** Colony formation assays were performed over ten days of cell culture. The number and size of colonies were quantified; n=4. Data are expressed as mean ± SD. ***p < 0.001. **Supplemental Figure 4.** PF-543 does not induce apoptosis in the liver. DEN-treated mice were administered with vehicle (veh) or PF-543 (PF) for 12 weeks. **A** Apoptosis in non-tumorous (NT) and tumorous (T) liver tissues were detected by cleaved caspase-3 immunohistochemistry; scale bar = 50 μm. **B** Levels of cleaved caspase-3 in liver tissues were examined by Western blotting. n = 9. **Supplemental Figure 5.** PF-543 does not alter CD31 expression in endothelial cells. HUVECs and HMEC-1 cells were treated with 5 μM PF-543 (PF) for 16 h. CD31 protein levels were determined by Western blotting. **Supplemental Figure 6.** PF-543 impairs angiogenesis in HMEC-1 cells. HMEC-1 cells were treated with PF-543 at the indicated concentrations for 16 h. **A** Tube formation was induced by 50 ng/ml VEGF-A. Quantification of the tube formation is presented as the number of junctions, segment length and total branching length. kpx, 1000 pixels; scale bar = 100 μm; n = 3. **B** Cell migration was determined by transwell assay, and migrated cells were stained and quantified with crystal violet; n = 3. **C** Cell viability was determined by MTS assay; n = 4. **D** Cell death was determined using flow cytometry with propidium iodide (PI) staining; PI-, living cells; PI+, dead cells; n = 3. Data are expressed as mean ± SD. ***p < 0.001. **Supplemental Figure 7.** Network Visualization of Glycolysis and VEGF Signaling Interactions. Genes associated with glycolysis and VEGF signaling pathways were shortlisted from KEGG datasets. The subnetwork was generated by pathway commons analysis, with dotted lines representing known regulatory relationships, physical interactions, and expression correlations among the genes. Node size is proportional to the degree of connectivity within this subnetwork. **Supplemental Figure 8.** PF-543 reduces PFKFB3 expression in liver tumors. DEN-injected mice were treated with vehicle (veh) or PF-543 (PF) for 12 weeks. Immunofluorescent staining of CD31 (green) and PFKFB3 (red), with DAPI (blue) counterstaining, was conducted in non-tumorous (NT) and tumorous (T) liver tissues; scale bar = 20 μm. **Supplemental Figure 9.** PF-543 elevates PFKFB3 mRNA expression in HUVECs. Primary HUVECs were treated with 5 μM PF-543 for 16 h. mRNA levels of PFKFB3 were determined by qRT-PCR, relative to β-actin; n = 3. Data are expressed as mean ± SD. ***p < 0.001. **Supplemental Figure 10.** Phospho-SphK1 and PFKFB3 levels are correlated in human HCC. **A** PFKFB3 levels were examined using immunohistochemical staining in tumorous (T) and para-tumorous (para-T) tissues of human HCC specimens; scale bar = 50 μm; n = 10. **B** Non-parametric Spearman correlations between phospho(p)-SphK1, total SphK1 and PFKFB3 expression were analyzed in tumorous tissues. *p* < 0.05 is considered significant.

## Data Availability

All data and materials supporting the conclusion of this study are available from the corresponding author upon reasonable request.
