# Supplementary material for: Targeting the SphK1/S1P/PFKFB3 axis suppresses hepatocellular carcinoma progression by disrupting glycolytic energy supply that drives tumor angiogenesis
Source: J Transl Med. 2024 Jan 10;22:43. doi: 10.1186/s12967-023-04830-z (PMC10782643; doi:10.1186/s12967-023-04830-z)
Supplement: Supplementary file 1 — Additional file 1. Supplemental Figure 1. PF-543 specifically reduces hepatic S1P levels in DEN-treated mice. Hierarchical clustering heatmap contains all identified sphingolipid species. The clustering dendrogram was determined based on the Euclidean distances between lipid variables using the Ward clustering method. Color keys indicate standardized lipid concentrations in Z-scores, with red denoting higher and blue denoting lower concentrations. ***, p < 0.001; n = 6. Supplemental Figure 2. PF-543 decreases plasma S1P levels in DEN-treated mice. Plasma levels of ceramide (Cer), sphingosine (Sph), and S1P were analyzed using lipidomics. Data are expressed as mean ± SD; n = 9. ***p < 0.001. Supplemental Figure 3. PF-543 exhibits minimal cytotoxicity in Huh7 HCC cells. A Huh7 cells were treated with 5 μM PF-543 (PF) for 16 h, prior to the measurement of ceramide (Cer), sphingosine (Sph) and S1P levels by lipidomics; n = 4. B Cell viability was determined using MTS assay; n = 6. C Cell death was assessed using flow cytometry with propidium iodide (PI) staining; PI-, living cells (green); PI+, dead cells (red); n = 3. D Colony formation assays were performed over ten days of cell culture. The number and size of colonies were quantified; n=4. Data are expressed as mean ± SD. ***p < 0.001. Supplemental Figure 4. PF-543 does not induce apoptosis in the liver. DEN-treated mice were administered with vehicle (veh) or PF-543 (PF) for 12 weeks. A Apoptosis in non-tumorous (NT) and tumorous (T) liver tissues were detected by cleaved caspase-3 immunohistochemistry; scale bar = 50 μm. B Levels of cleaved caspase-3 in liver tissues were examined by Western blotting. n = 9. Supplemental Figure 5. PF-543 does not alter CD31 expression in endothelial cells. HUVECs and HMEC-1 cells were treated with 5 μM PF-543 (PF) for 16 h. CD31 protein levels were determined by Western blotting. Supplemental Figure 6. PF-543 impairs angiogenesis in HMEC-1 cells. HMEC-1 cells were treated with PF-5 [file 12967_2023_4830_MOESM1_ESM.pdf]

Supplemental Figure 1

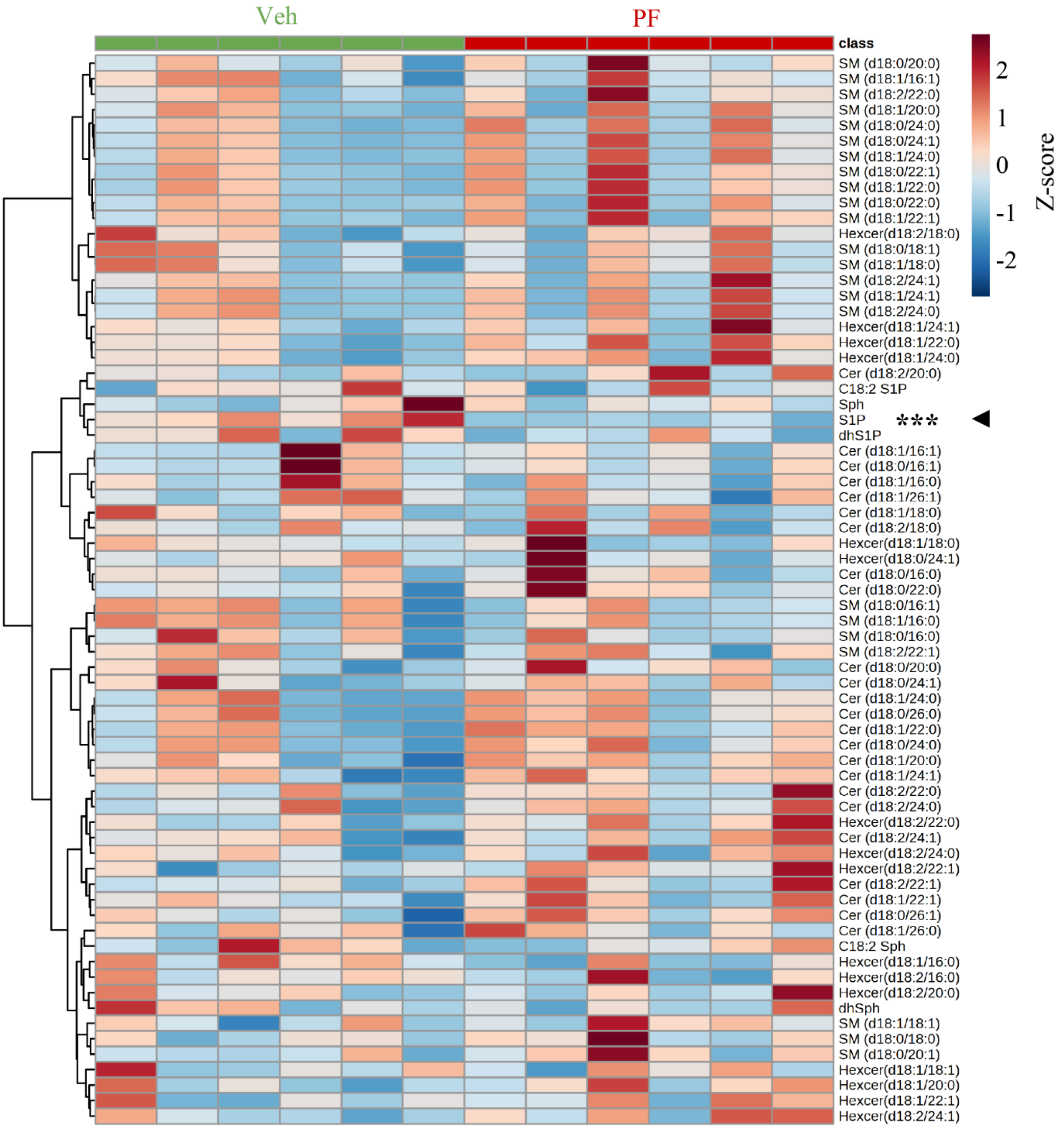

Supplemental Figure 1. PF-543 specifically reduces hepatic S1P levels in DEN-treated mice. Hierarchical clustering heatmap contains all identified sphingolipid species. The clustering dendrogram was determined based on the Euclidean distances between lipid variables using the Ward clustering method. Color keys indicate standardized lipid concentrations in Z-scores, with red denoting higher and blue denoting lower concentrations. ***, $p<0.001$; n=6.

Supplemental Figure 2

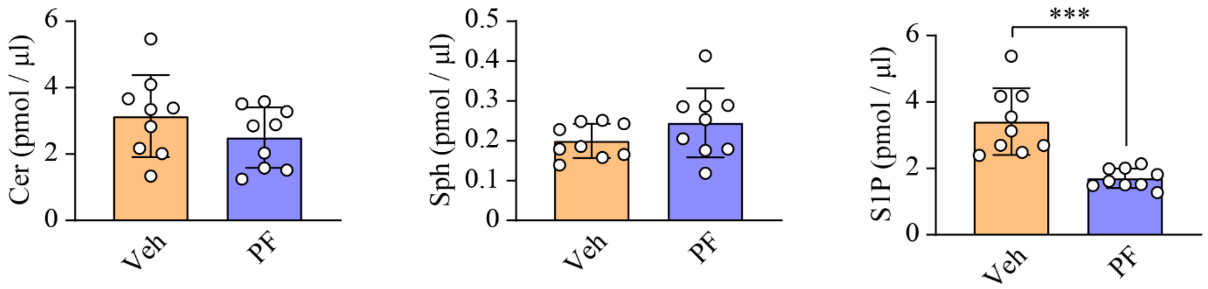

Supplemental Figure 2. PF-543 decreases plasma S1P levels in DEN-treated mice. Plasma levels of ceramide (Cer), sphingosine (Sph), and S1P were analyzed using lipidomics. Data are expressed as mean ± SD; n=9. ***p<0.001.

Supplemental Figure 3

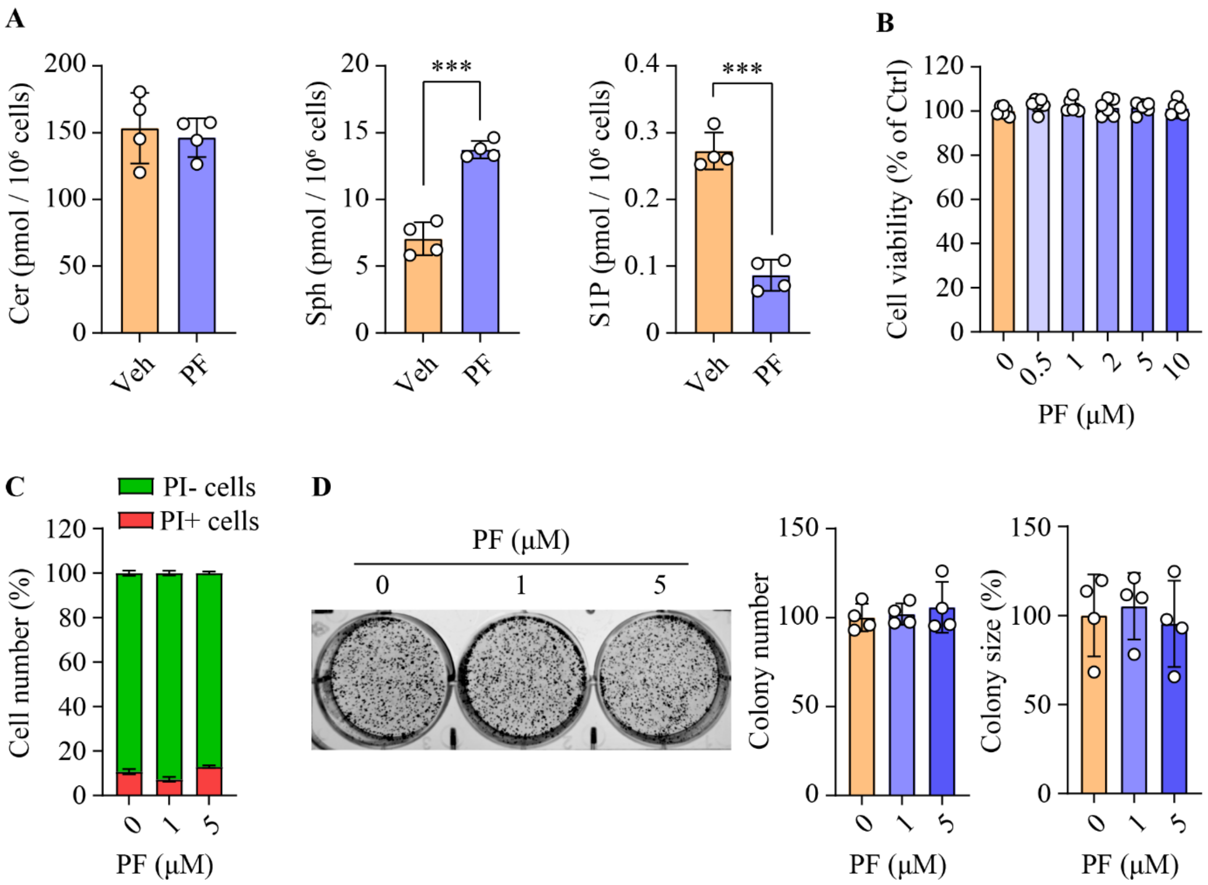

Supplemental Figure 3. PF-543 exhibits minimal cytotoxicity in Huh7 HCC cells. A, Huh7 cells were treated with 5 μM PF-543 (PF) for 16 h, prior to the measurement of ceramide (Cer), sphingosine (Sph) and S1P levels by lipidomics; n=4. **B,** Cell viability was determined using MTS assay; n=6. **C,** Cell death was assessed using flow cytometry with propidium iodide (PI) staining; PI-, living cells (green); PI+, dead cells (red); n=3. **D,** Colony formation assays were performed over ten days of cell culture. The number and size of colonies were quantified; n=4. Data are expressed as mean ± SD. ***p<0.001.

Supplemental Figure 4

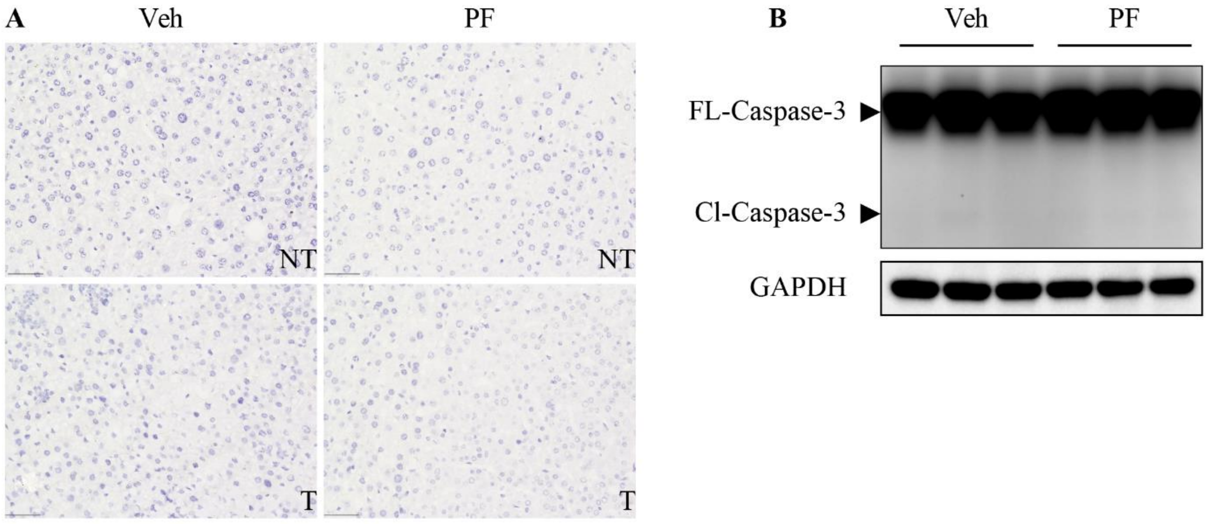

Supplemental Figure 4. PF-543 does not induce apoptosis in the liver. DEN-treated mice were administered with vehicle (veh) or PF-543 (PF) for 12 weeks. **A,** Apoptosis in non-tumorous (NT) and tumorous (T) liver tissues were detected by cleaved caspase-3 immunohistochemistry; scale bar = 50 μm. **B,** Levels of cleaved caspase-3 in liver tissues were examined by Western blotting. n=9.

Supplemental Figure 5

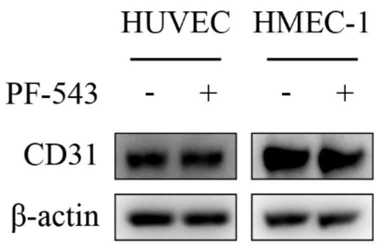

Supplemental Figure 5. PF-543 does not alter CD31 expression in endothelial cells. HUVECs and HMEC-1 cells were treated with 5 µM PF-543 (PF) for 16 h. CD31 protein levels were determined by Western blotting.

Supplemental Figure 6

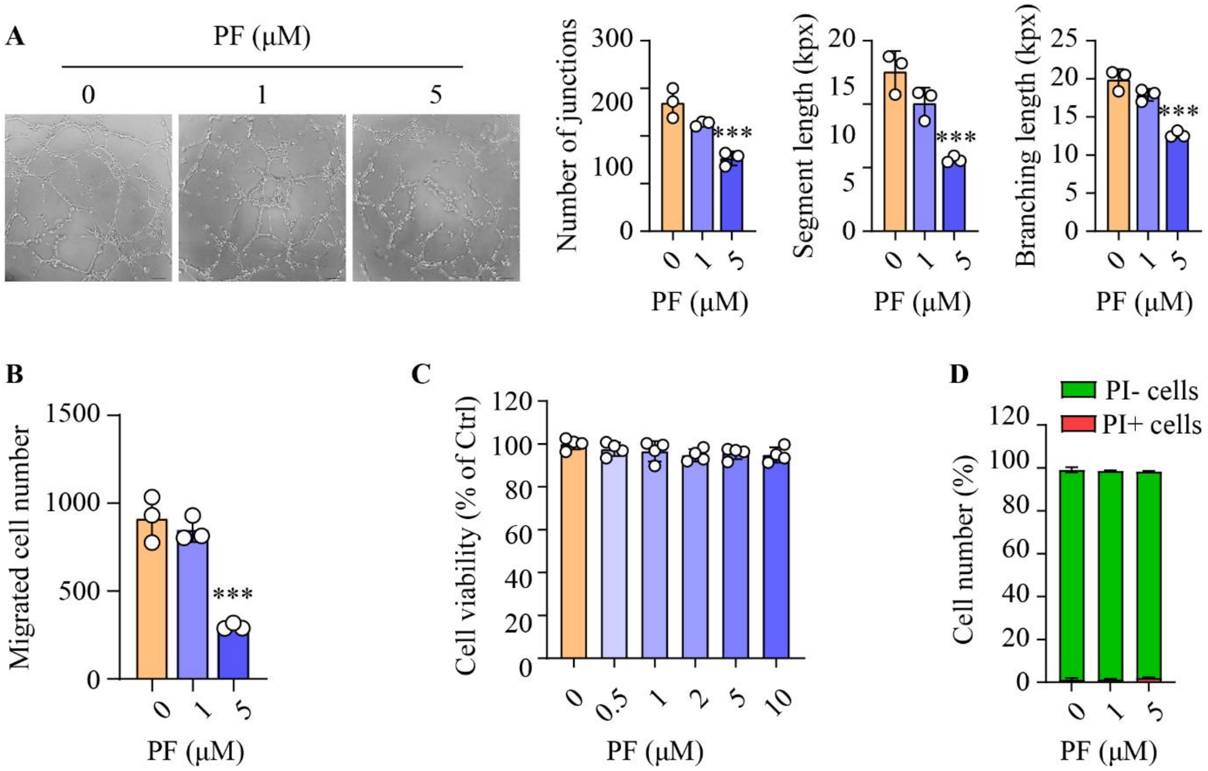

Supplemental Figure 6. PF-543 impairs angiogenesis in HMEC-1 cells. HMEC-1 cells were treated with PF-543 at the indicated concentrations for 16 h. **A,** Tube formation was induced by 50 ng/ml VEGF-A. Quantification of the tube formation is presented as the number of junctions, segment length and total branching length. kpx, 1000 pixels; scale bar = 100 µm; n=3. **B,** Cell migration was determined by transwell assay, and migrated cells were stained and quantified with crystal violet; n=3. **C,** Cell viability was determined by MTS assay; n=4. **D,** Cell death was determined using flow cytometry with propidium iodide (PI) staining; PI-, living cells; PI+, dead cells; n=3. Data are expressed as mean ± SD. ***p<0.001.

Supplemental Figure 7

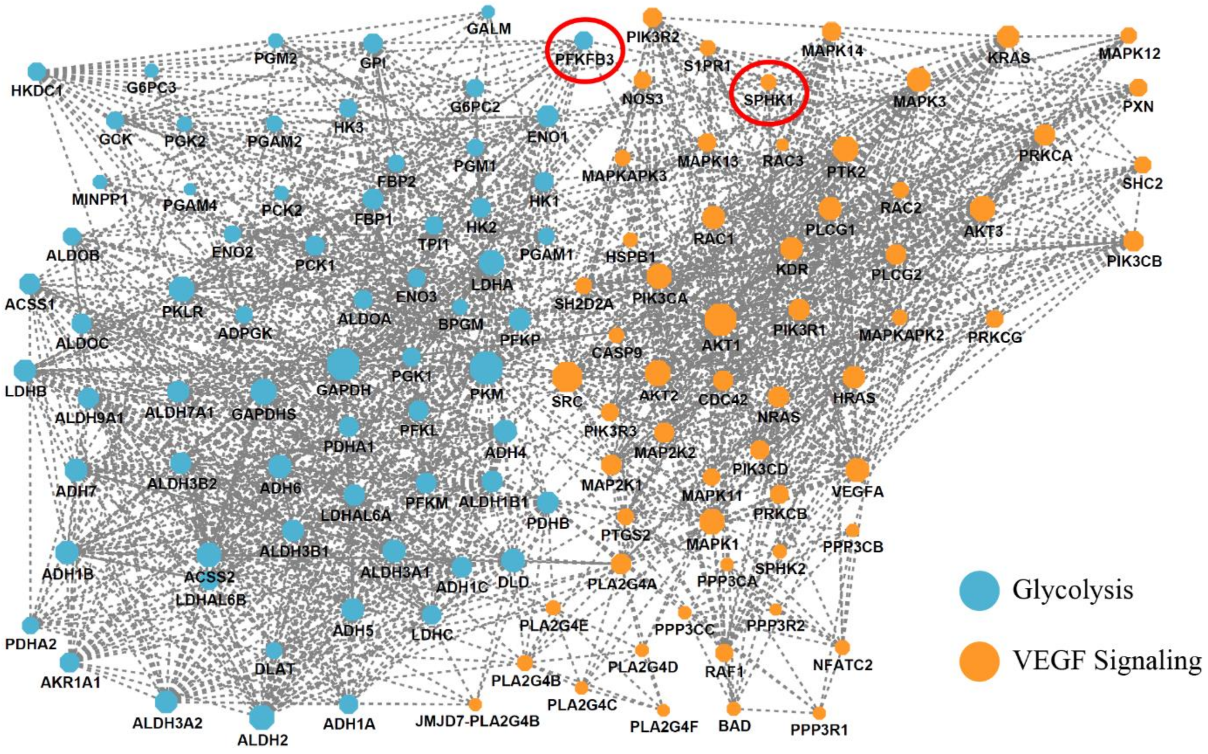

Supplemental Figure 7. Network Visualization of Glycolysis and VEGF Signaling Interactions. Genes associated with glycolysis and VEGF signaling pathways were shortlisted from KEGG datasets. The subnetwork was generated by pathway commons analysis, with dotted lines representing known regulatory relationships, physical interactions, and expression correlations among the genes. Node size is proportional to the degree of connectivity within this subnetwork.

Supplemental Figure 8

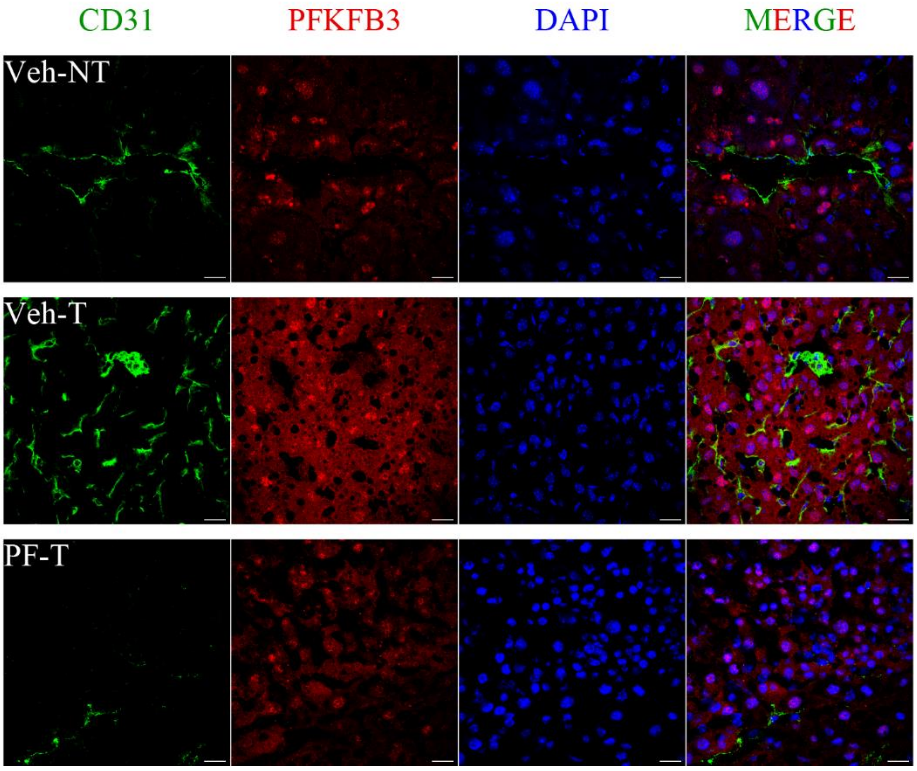

Supplemental Figure 8. PF-543 reduces PFKFB3 expression in liver tumors. DEN-injected mice were treated with vehicle (veh) or PF-543 (PF) for 12 weeks. Immunofluorescent staining of CD31 (green) and PFKFB3 (red), with DAPI (blue) counterstaining, was conducted in non-tumorous (NT) and tumorous (T) liver tissues; scale bar = 20 μm.

Supplemental Figure 9

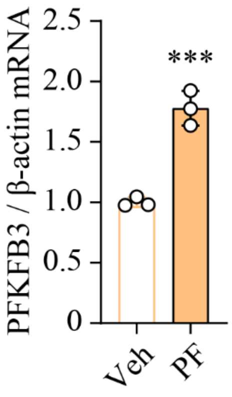

Supplemental Figure 9. PF-543 elevates PFKFB3 mRNA expression in HUVECs. Primary HUVECs were treated with 5 μM PF-543 for 16 h. mRNA levels of PFKFB3 were determined by qRT-PCR, relative to β-actin; n=3. Data are expressed as mean ± SD. ***p<0.001.

Supplemental Figure 10

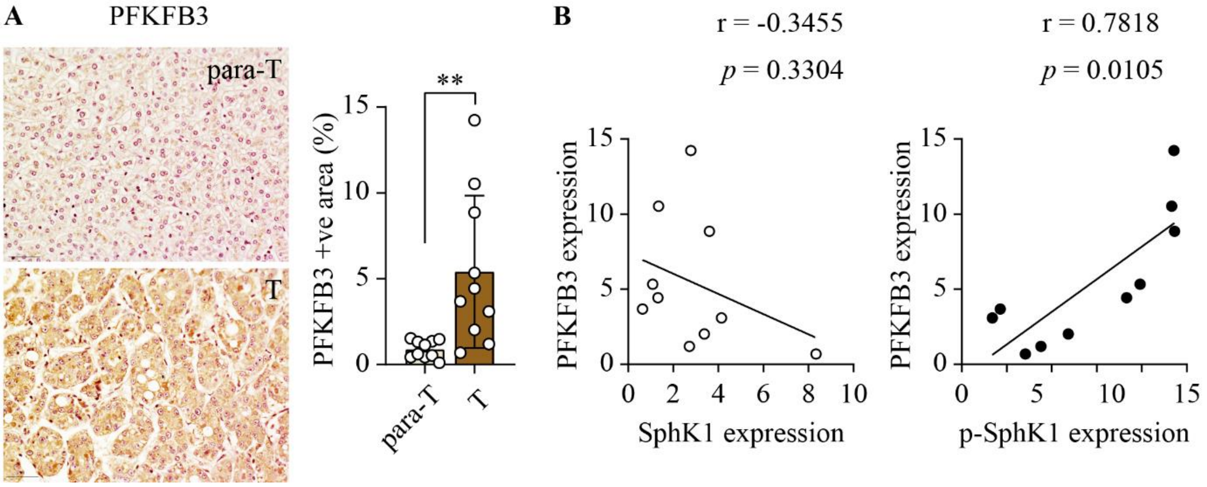

Supplemental Figure 10. Phospho-SphK1 and PFKFB3 levels are correlated in human HCC. A, PFKFB3 levels were examined using immunohistochemical staining in tumorous (T) and para-tumorous (para-T) tissues of human HCC specimens; scale bar = 50 μm; n=10. **B,** Non-parametric Spearman correlations between phospho(p)-SphK1, total SphK1 and PFKFB3 expression were analyzed in tumorous tissues. $p<0.05$ is considered significant.
